# A Bird's-Eye View of Exercise Intervention in Treating Depression Among Teenagers in the Last 20 Years: A Bibliometric Study and Visualization Analysis

**DOI:** 10.3389/fpsyt.2021.661108

**Published:** 2021-06-18

**Authors:** Yanwei You, Dizhi Wang, Yuning Wang, Zhipeng Li, Xindong Ma

**Affiliations:** Division of Sport Science and Physical Education, Tsinghua University, Beijing, China

**Keywords:** exercise intervention, depression, teenagers, bibliometric, visualization analysis

## Abstract

**Background:** Exercise is medicine. Multiple studies on the effects and mechanisms of exercise in treating depression among teenagers and adolescents have been widely reported. However, literature involving scientometric analysis of this topic is sparse. Here, we endeavored to conduct a bibliometric study and visualization analysis to give a bird's-eye view of publications between 2000 and 2020 on exercise therapy treating depression.

**Methods:** Relevant original publications were obtained from the Science Citation Index Expanded in the Web of Science Core Collection (WoSCC) database between 2000 and 2020. CiteSpace (5.7.R 5) and VOSviewer (1.6.16) software were used to perform bibliometric analysis of countries, institutions, categories, journals, authors, references, and keywords involved in this topic.

**Results:** A total number of 975 articles on this field were retrieved from the WoSCC database and we identified an overall increase in the amount of publications over the past two decades, with the United States and Harvard University leading the field. Most related publications were published in the journals with a focus on sport, medicine, rehabilitation, psychology, and health, as represented by the dual-map overlay. A series of authors and co-cited authors were identified as main contributors in the exercise-depression-teenager domain. Three major clusters were explored based on the reference co-citation analysis: “exercise,” “suicide,” and “concussion”.

**Conclusions:** Current concerns and hotspots of exercise intervention in depression treatments were summarized by “individual level,” “social level,” “role of exercise,” and “research quality.” We considered that the following four directions were potential future perspectives: “research on the effect of specific exercise intervention,” “research on the essence of exercise and sports,” “research on the combination mode of ‘exercise + X',” and “research on the micro and molecular level,” which should receive more attention.

## Introduction

Depression is one of the most common psychiatric conditions (1–5) and is becoming a leading cause of global health burden (6–8). According to the research results from the World Health Organization, over 300 million people, or 4.4% of the global population, suffer from depression (9). As a chronic medical illness that can affect both physical and psychological health (10, 11), depression is characterized by sadness, insomnia or hypersomnia, absence of enjoyment, loss of interest, lack of energy, and an inability to be optimistic and positive toward life. Depressive disorder in teenager groups is a non-negligible psychiatric disorder that remains to be solved. Studies also showed that the 1 year prevalence rate of adolescent depression was estimated to be 5.6 and 2.8% for girls and boys below the age of 13 (12, 13). Adolescence is a time of increasing vulnerability for poor mental health, and so-called “puberty blues” may disturb teenagers' social functioning, familial relationships, and personality cultivation. A previous research indicated that at least one-quarter of young populations might experience an episode of depressive disorder before 19 years of age (14). Given the considerable scope and influence of depression in youth groups, there is a pressing need to identify efficient and convenient strategies to deal with these issues caused by depressive disorders.

However, there are deficiencies in approved pharmacological therapy options in young populations partly due to unknown risks associated with lasting effects during their future life. Hence, interest in non-pharmacological methods is now gaining popularity with the development of complementary or integrated medicine strategies. Exercise is considered to be a potential strategy for the prevention, treatment, and rehabilitation of depression on a wide scale. Early-life training of appropriate exercise may present a distinctive “window of opportunity” (15) to induce long-term beneficial effects in young individuals, including but not limited to the treatment of depression. Generally, there is an increasing body of meta-analysis-based studies (16–20) suggesting that physical activity (PA) and exercise are useful methods for improving depressive symptoms, promoting health outcomes, and preventing other mood disorders.

Bibliometric analysis is an informational process which involves mathematics and statistics tools to identify publication entities, development tendencies, and focuses of research subjects or domains (21, 22). Based on multiple indexes like references, authors, journals, countries, and institutions, it can conduct an in-depth assessment of the topic trends and the focus of a certain field (23–25). The results of bibliometric analysis are accessible and convenient for calculating the productivity of authors and institutions, identifying geographic distributions, uncovering the knowledge map, and then providing advice for future research and decision making. Compared with the traditional systematic review and meta-analysis, bibliometric analysis can reveal the current situation and evolution of a research topic from a more systematic and intuitive level. The common difficulties in the existing meta-analyses of exercise intervention for adolescent depression are that the number of included publications are not enough and the outcome indicators are quite different. Therefore, the high heterogeneity and risk bias make it difficult for readers to have a comprehensive grasp of this topic. However, by providing visual graphics, a bibliometric review can help investigators clearly understand the past, present, and future of this domain. Results from bibliometric analysis can also help researchers to identify the current concerns and hot spots, thereby suggesting ideas and perspectives to guide future research directions. In view of this, applying the bibliometric method has outstanding significance and extreme importance in the field of exercise intervention in treating adolescent depression.

A variety of software is available that can be used for bibliometric studies, such as, VOSviewer (26, 27) and CiteSpace (28, 29). VOSviewer is a freely available computer program that is often applied to construct visualization maps (30, 31), using the full details of indexes such as authors, journals, keywords, or any other network data. Besides, the visualization function of VOSviewer also allows scholars to excavate valuable information by data-mining technology and display it intuitively. CiteSpace is a Java-based scientific mapping software for scientometric and comparative analysis. By presenting numerous data in the form of knowledge maps, results including productivity of authors and institutions, geographic distribution of regions, and cooperative relations can be presented directly to reflect the development of a certain field (32, 33).

Although previous literature has provided an overview of several theoretical and empirical domains on the relationship between exercise and depression in adolescents, to the best of our knowledge, none of these publications made use of a quantitative and visualization approach to survey the longitudinal and transversal characteristics, developments, and multiple ramifications of this topic. It should be noted that this review does not illustrate exercise prescriptions for specific depressive symptoms in adolescents, but presents a visual analysis to evaluate research status and discover the potential mechanisms and applicable strategies of exercise intervention for depression. We would like to identify collaboration networks among authors, institutions, and countries, and explore crucial contributors in this field since the beginning of the new century. In addition, we aimed to investigate which topics of interests were recent hot spots and had potential to be prominent in the up-coming years. To this end, we constructed this organic image of exercise treatment of depression in teenagers to portray a bottom-up view in a historical and prospective standpoint, thereby shedding new light for scholars to help them draft and manage their scientific research.

## Materials and Methods

### Data Acquisition and Search Strategy

A comprehensive search was conducted using the Science Citation Index Expanded in the Web of Science Core Collection (WoSCC) database on March 29, 2021, at Tsinghua University, Beijing, China. The reason for using the SCI-E of WoSCC was due to its coverage of numerous records and documents, especially in the domains of psychiatry and mental health. This database has been used as a data resource in some previous bibliometric studies (34, 35). To define the time interval of this research, we set the time span from January 1, 2000 to December 31, 2020. To guarantee the representativeness of the internalized literature, the document types were limited to “article” or “review” and the language was limited to English. We used the “title,” “abstract,” and “author keywords” filters at the same time to ensure the relevance of the included literature. The search strategy was as follows: topic = (exercise OR fitness OR sports OR sport OR physical exercise OR exercise training OR physical activity OR physical fitness OR exercise therapy OR aerobic exercise OR non-aerobic exercise OR resistance exercise OR strength exercise OR breathing exercises OR muscle stretching exercises OR walking OR jogging OR running OR cycling OR swimming OR weight lifting OR tai chi OR qigong OR yoga) AND (depression OR depress OR dysthymia OR manic depress OR major depress OR clinical depress OR depressive disorder OR depressive symptom) AND (adolescents OR adolescent OR teens OR teen OR teenagers OR teenager OR youth OR youths OR female adolescents OR female adolescent OR male adolescent OR male adolescents). To avoid bias, all hits were retrieved as “full record and cited references” files from WoSCC on March 29, 2021 for further analysis.

### Analysis Tools

CiteSpace (5.7.R 5 Version) was applied to conduct bibliometric analysis on the literature, including key features such as publication categories, countries/regions, organizations, journals, references, and keywords. Dual-map overlays of journals and burst strength analysis were also explored by CiteSpace. The parameters of CiteSpace were set as follows: link retaining factor = 2, look back years = −1, e for top *N* = 2, time span = 2000–2020, years per slice = 1, selection criteria = top 50. After a first visualization of the network, the pathfinder function was applied to use a link reduction algorithm (36), which can provide a more reasonable and precise network configuration (37). VOSviewer (1.6.16) was used to generate and analyze authors and keywords. The parameters of VOSviewer were set as follows: counting method (fractional counting) and “ignore documents with a large number of authors” (maximum number of authors per document is 25) (38).

Different nodes in a map represented indexes including a country, institution, or journal. The size of the nodes spoke on behalf of the centrality of publications or frequency, and a node with large size typically indicated high occurrence or citation frequency as a pivotal point (39, 40). The links between nodes showed the network of cooperation, collaboration, or co-citation, and the color of nodes and links indicated different clusters (41). The clustering function was used to find the major groups in which single nodes could be divided into. Occurrence burst expressed a term that occurred frequently during a given period of time which can be regarded as emerging foci or frontiers, and the burst of nodes was developed with a given network through Kleinberg's algorithm (42). Microsoft Excel 2016 software was applied to describe and predict the annual output of related publications. The function model was laid out as quadratic polynomial: *f(x)* = *ax*^2^ + *bx* + *c*, in which *x* represented the year of publication and *f(x)* demonstrated the number of publications. The reason for using quadratic polynomial instead of linear, exponential, logarithmic, or other functions was that the polynomial can better reflect the relationship between publication number and time. Through the above approaches, we have a profound understanding of the historical trend, present situation, and future forecast of exercise intervention targeting depressive disorders among teenagers.

## Results

### Annual Outputs and Growth Trend

**Figure 1:**
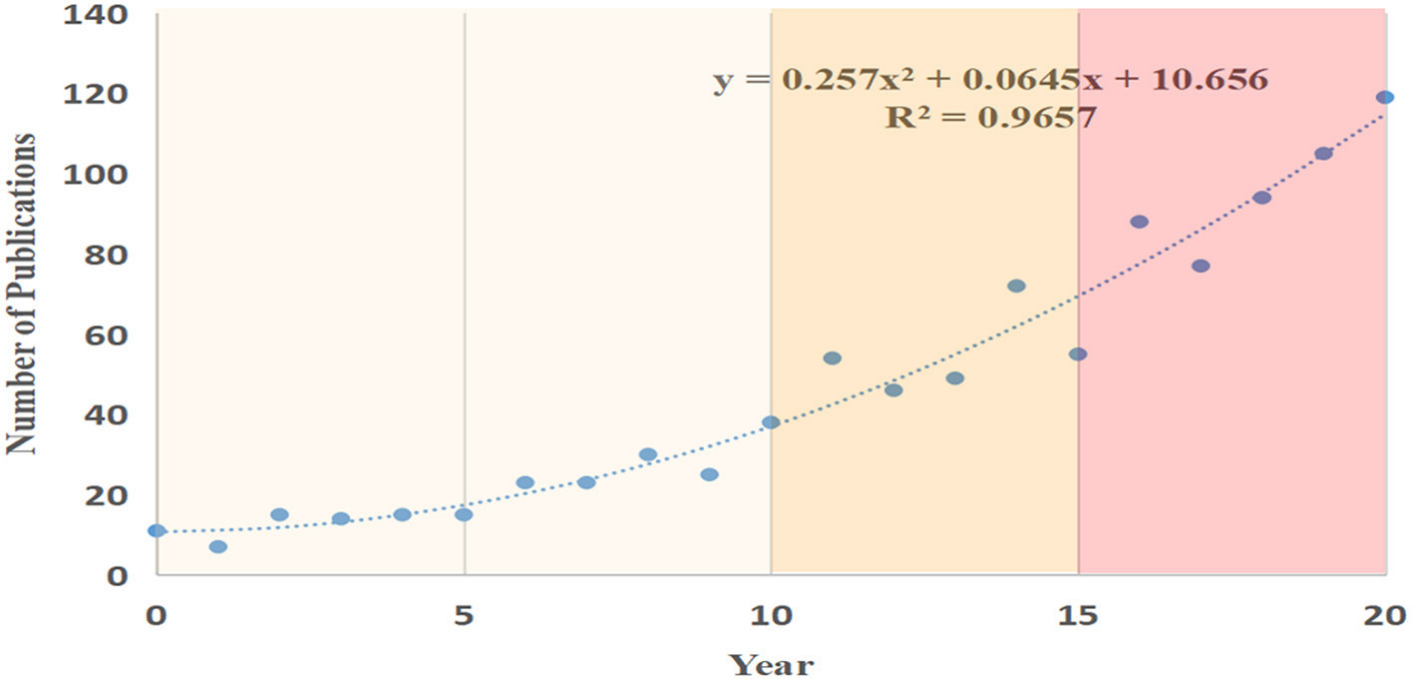
The output of publications and growth trend in exercise-depression-teenager research.

In total, we retrieved 975 exercise-depression-teenager-related research published between 2000 and 2020, the average annual output was 46. The relationship between the amount of documents per year and the publication amount is showed in Figure 1. In 2000, only 11 works were published; however, two decades later in 2020, the annual output reached 119, almost 10 times the previous amount. The annual output showed an overall upward tendency although with some fluctuations during the time interval from 2000 to 2020.

Publications in the exercise-depression-teenager field can be divided into three stages: the initial stage (2000–2010), second stage (2011–2015), and third stage (2016–2020). Before the year 2010, no more than 40 papers were published annually. While after 2011, annual published research had increased sharply, and by 2015, the amount had reached more than 50 per year. After 2016, the number of publications rose exponentially, tripling in just 5 years to nearly 120 by the end of 2020. A growth trend model (*R*^2^ = 0.9657) predicted that the amount of literature related to exercise treating depression in adolescents would be progressive and promising in the near future.

### Countries and Institutions

Scholars from more than 70 regions and 430 institutions contributed to publications on the field of exercise-depression-teenager research. The details of the top 10 countries and institutions are listed in Table 1.

**Table 1:** Ranking of top 10 countries and institutions in the field of exercise-depression-teenager research from 2000 to 2020.

**Rank**	**Country**	**Publications**	**Centrality**	**Institution**	**Publications**	**Centrality**
1	United States	406	0.65	Harvard Univ	32	0.17
2	Canada	117	0.11	Univ Toronto	24	0.08
3	England	114	0.39	Univ Montreal	19	0.07
4	Australia	96	0.06	Univ Melbourne	19	0.05
5	China	71	0.03	Univ Penn	19	0.07
6	Netherlands	40	0.04	Kings Coll London	18	0.07
7	Sweden	38	0.09	Univ Washington	18	0.05
8	Germany	32	0.08	Univ Ottawa	16	0.01
9	Italy	26	0.01	UCL	15	0.04
10	Norway	24	0.02	Univ Calgary	13	0.03
10^*^				Karolinska Inst	13	0.04
10^*^				Columbia Univ	13	0.03

* *Indicates a tie for 10th place*.

**Figure 2:**
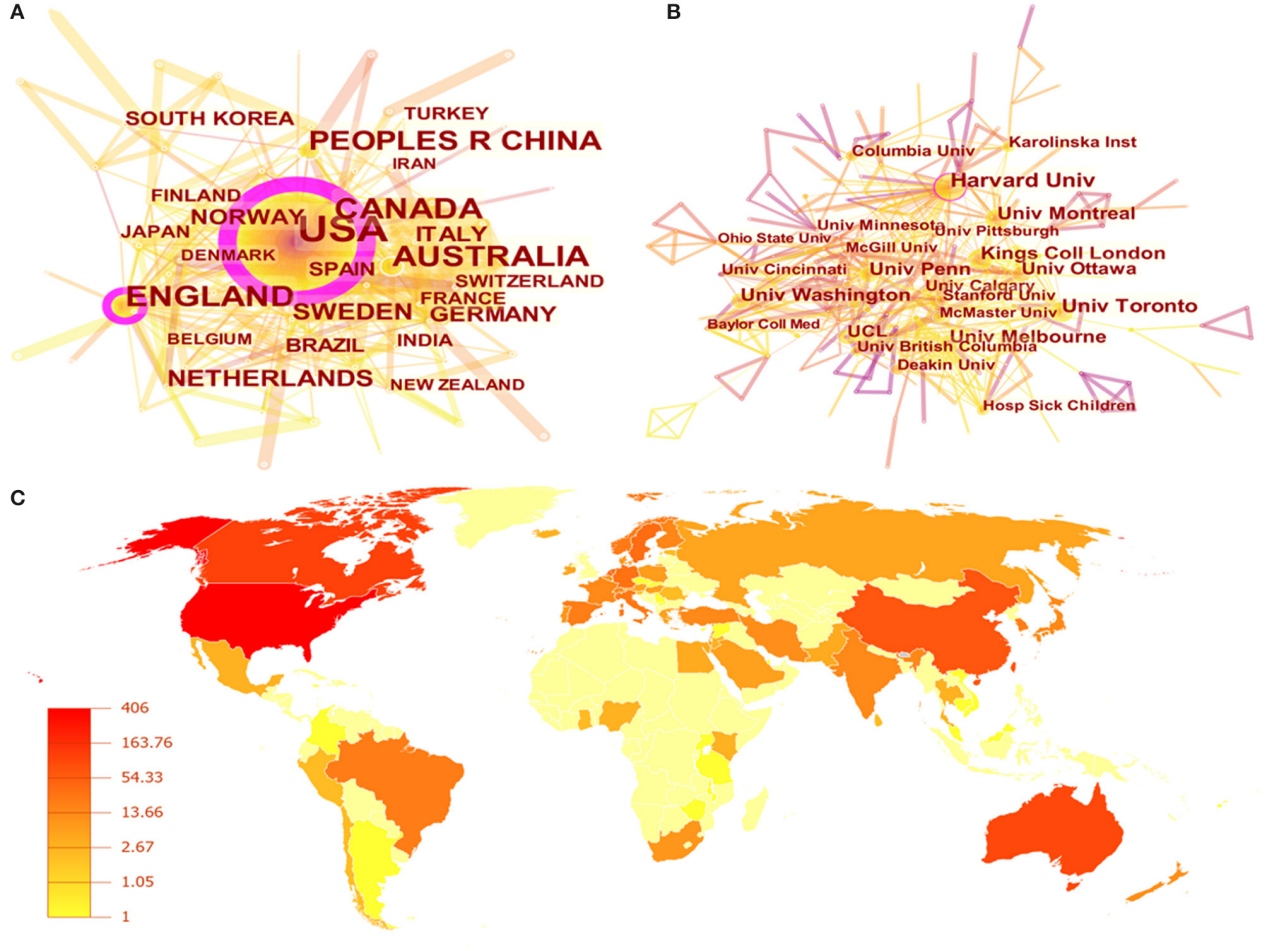
The collaboration of countries and institutions. **(A)** Map of countries with publications in exercise-depression-teenager research. **(B)** Map of institutions with publications in exercise-depression-teenager research. **(C)** The distribution of countries/regions engaged in exercise-depression-teenager research.

As shown in Figure 2A, the cooperation conditions were generated by CiteSpace software with 73 nodes and 336 links, and close relationships were observed among these countries. Nine of the top 10 prolific countries were developed countries, while China was the only developing country. This implied that developed regions may input more resources into anti-depression strategies and pay more attention to adolescent health. The United States ranked first and had the highest amount of literature, 406, also the highest centrality (0.65). Canada was the second most productive country with 117 publications, followed by England (114 publications), Australia (96 publications), and China (71 publications). The top five countries' contributions were all above 70 publications, which indicated that they contributed chiefly in research achievements.

As shown in Figure 2B, the collaborations between institutions were presented with 437 nodes and 757 links simultaneously. According to the definition of links and nodes, these organizations had close ties with each other and have a considerable academic impact. Harvard University participated in the most studies (*n* = 32), followed by the University of Toronto (*n* = 24), University of Montreal (*n* = 19), University of Melbourne (*n* = 19), and University of Pennsylvania (*n* = 19). Furthermore, all of the top 10 institutions belonged to Western nations, also indicating that Western institutions occupied the first-tier position in the field of exercise-depression-teenager research. The distribution of these countries is presented in Figure 2C. From this figure, we can also find that countries and institutions in North America, western Europe, Australia, and East Asia led the way in the field of exercise-depression-teenager research.

### Categories and Journals

**Figure 3:**
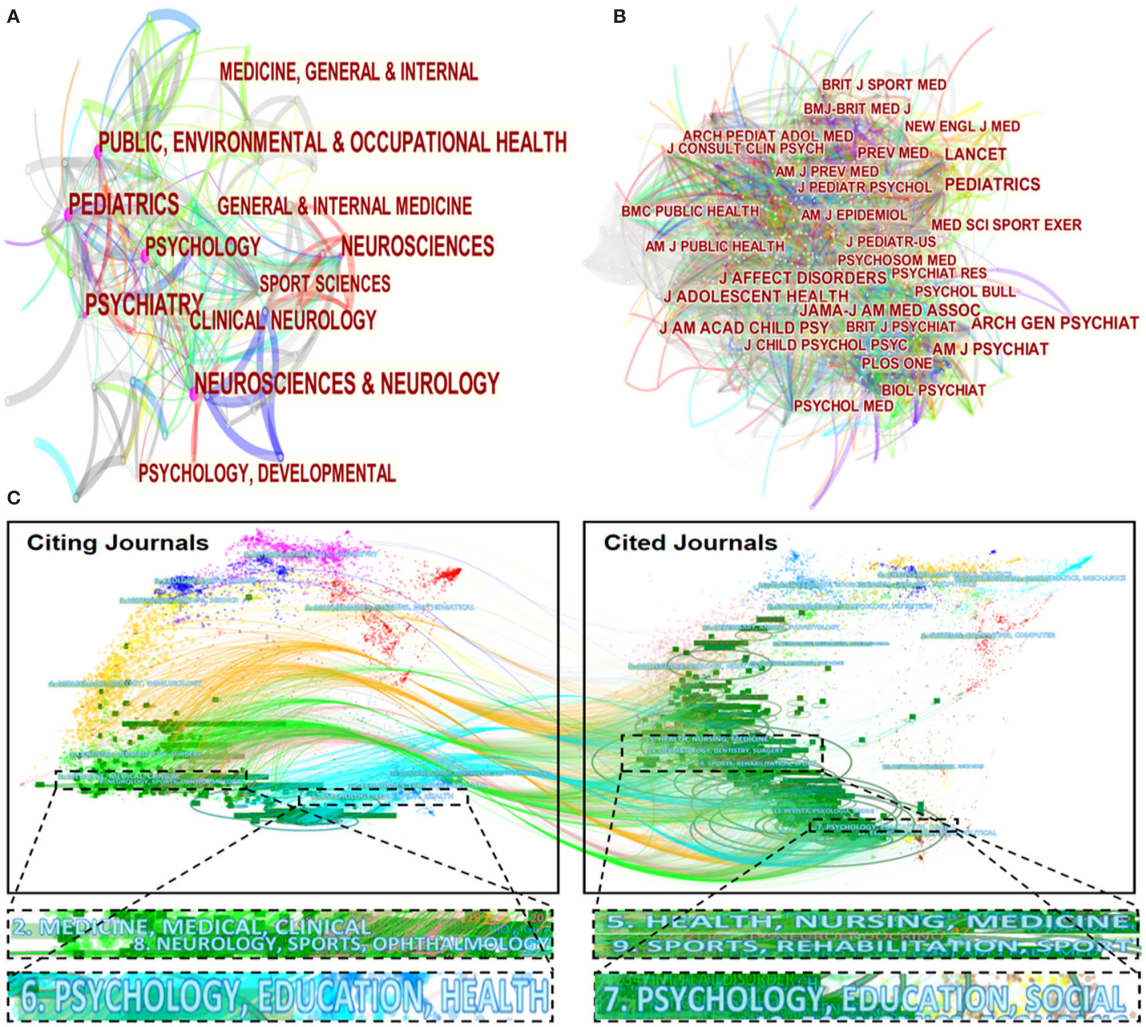
The distribution of categories and journals. **(A)** Map of categories with publications in exercise-depression-teenager research. **(B)** Map of journals with publications in exercise-depression-teenager research. **(C)** Dual-map overlay of journals that published work related to exercise-depression-teenager research.

The most related categories in publishing studies concerned with exercise and depression in young groups are presented in Figure 3A. Categories including *Psychology, Psychiatry, Public Environmental and Occupational Health, Pediatrics*, and *Neurosciences and Neurology* seemed to be the most common. It was noteworthy that *Sport Sciences* also played a significant role in the formation of discipline composition. The merged network of nodes was 89, and links accounted for 341. Based on the analysis of the multidisciplinary composition of the categories involved in this field, we found that “cross-disciplinary communities” were formed. The various research directions of different categories were gathered together, which represented the diversity and richness of the publication sources.

In all, 428 journals published articles on exercise-depression-teenager research. As shown in Table 2, the top 10 citing journals contributed a total of 206 publications, making up nearly 20% of all the 975 documents retrieved. The *JOURNAL OF ADOLESCENT HEALTH* (IF 2019 = 3.900) published the greatest amount of literature (44 publications, 4.51%), followed by *BMC PUBLIC HEALTH* (26 publications), *JOURNAL OF AFFECTIVE DISORDERS* (23 publications), and *PLOS ONE* (23 publications). In terms of impact factor (IF), all 10 journals were ranked from 1.673 to 7.890, only two journals' impact factor exceeded 3, whereas the average value of these 10 journals' impact factors was 3.771.

**Table 2:** Ranking of top 10 journals and co-cited journals in the field of exercise-depression-teenager research from 2000 to 2020.

**Rank**	**Citing journal**	**Publications**	**Percentage (%)**	**IF (2019)**	**Cited journal**	**Co-citation counts**
1	JOURNAL OF ADOLESCENT HEALTH	44	4.51%	3.900	PEDIATRICS	370
2	BMC PUBLIC HEALTH	26	2.67%	2.521	ARCH GEN PSYCHIAT	344
3	JOURNAL OF AFFECTIVE DISORDERS	23	2.36%	3.892	J AM ACAD CHILD PSY	336
4	PLOS ONE	23	2.36%	2.740	J ADOLESCENT HEALTH	311
5	BMJ OPEN	16	1.64%	3.892	JAMA-J AM MED ASSOC	293
6	PEDIATRICS	15	1.54%	5.359	LANCET	280
7	PREVENTIVE MEDICINE	13	1.33%	3.788	J AFFECT DISORDERS	259
8	INTERNATIONAL JOURNAL OF ENVIRONMENTAL RESEARCH AND PUBLIC HEALTH	12	1.23%	2.849	AM J PSYCHIAT	249
9	JOURNAL OF SCHOOL HEALTH	12	1.23%	1.673	PSYCHOL MED	225
10	BEHAVIORAL BRAIN RESEARCH	11	1.13%	2.977	PLOS ONE	215
10^*^	COCHRANE DATABASE OF SYSTEMATIC REVIEWS	11	1.13%	7.890		

* *indicates a tie for 10th place*.

Figure 3B presents the relationships among cited journals. The merged network included 603 nodes and 5,827 links. *PEDIATRICS* took the lead with 370 co-citation counts, *ARCH GEN PSYCHIAT, J AM ACAD CHILD PSY*, and *J ADOLESCENT HEALTH* also contributed 344, 336, and 311, respectively. All these top 10 cited journals contributed more than 200 article co-citation counts, which represented that these journals were in the dominant position in citing exercise-depression-teenager-related works.

Figure 3C reveals a dual-map overlay of the research themes between citing journals and cited journals in the field of exercise-depression-teenager research. The citing journals are on the left side of the map while the cited journal are on the right. There are four citation paths in this picture. The grass green paths illustrate that studies published in “*medicine, medical, clinical*,” and “*neurology, sports, ophthalmology*” journals tended to cite journals primarily in the domains of “*health, nursing, medicine*” and “*sports, rehabilitation, sport*.” The paths colored with pale blue showcase that research published in “*psychology, education, health*” journals preferred to quote journals mostly in the domains of “*psychology, education, social*.”

### Authors and Co-Cited Authors

The top 10 authors, co-cited authors, and co-cited references of exercise-depression-teenager research are displayed in Table 3.

**Table 3:** Ranking of top 10 authors, co-cited authors, and co-cited references in the field of exercise-depression-teenager research from 2000 to 2020.

**Rank**	**Author**	**Counts**	**Co-cited author**	**Counts**	**Co-cited reference**	**Counts**
1	Ian Colman	8	Kessler, RC	167	Carter, T, 2016, J AM ACAD CHILD PSY, V55, P580, DOI 10.1016/j.jaac.2016.04.016	22
2	Serge Brand	7	Kovacs, M	122	Hoare, E, 2016, INT J BEHAV NUTR PHY, V13, P0, DOI 10.1186/s12966-016-0432-4	18
3	Gerber Markus	6	Radloef, LS	98	Bailey, AP, 2018, PSYCHOL MED, V48, P1068, DOI 10.1017/S0033291717002653	16
4	E. Holsboer-Trachsler	6	Beck, AT	92	McMahon, EM, 2017, EUR CHILD ADOLES PSY, V26, P111, DOI 10.1007/s00787-016-0875-9	16
5	Jennifer O'Loughlin	6	Lewinsohn, PM	87	Biddle, SJH, 2011, BRIT J SPORT MED, V45, P886, DOI 10.1136/bjsports-2011-090185	16
6	Anthony F Jorm	7	Vaeni, JW	84	Brown, HE, 2013, SPORTS MED, V43, P195, DOI 10.1007/s40279-012-0015-8	15
7	Brendon Stubbs	6	Birmaher, B	77	Jerstad, SJ, 2010, J CONSULT CLIN PSYCH, V78, P268, DOI 10.1037/a0018793	15
8	Jennifer Brunet	5	Costello, EG	77	Sund, AM, 2011, SOC PSYCH PSYCH EPID, V46, P431, DOI 10.1007/s00127-010-0208-0	13
9	Catherine M Sabiston	5	Sallis, JF	76	Maras, D, 2015, PREV MED, V73, P133, DOI 10.1016/j.ypmed.2015.01.029	13
10	Sergio D Iniguez	5	Biddle, SJH	76	Schuch, FB, 2016, J PSYCHIATR RES, V77, P42, DOI 10.1016/j.jpsychires.2016.02.023	11

**Figure 4:**
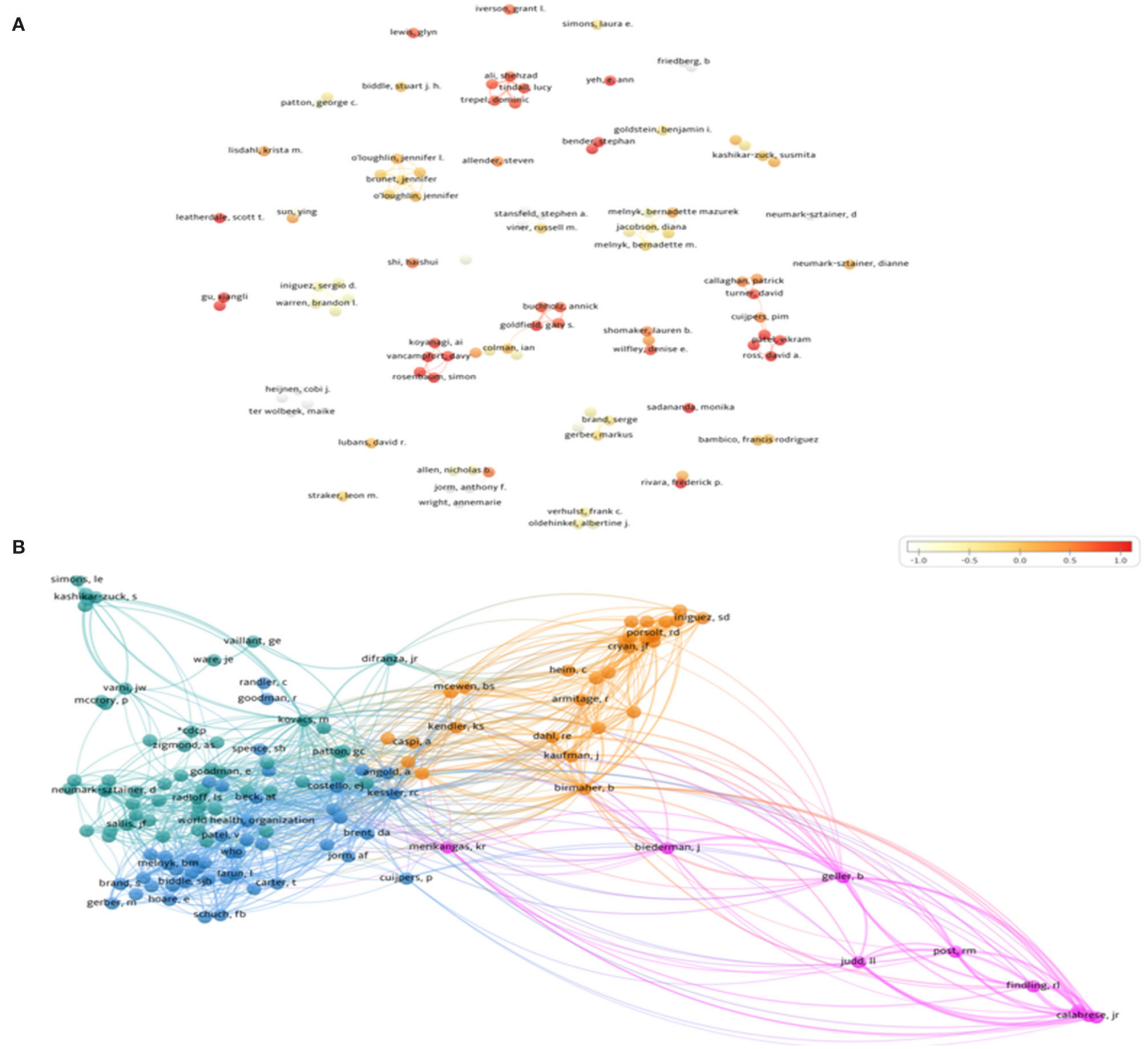
**(A)** The overlay visualization map of authors active in exercise-depression-teenager research based on the time of appearance. **(B)** The network visualization of co-cited authors active in exercise-depression-teenager research.

Discovery and exploration of remarkable authors and publications were carried out in this study. The tremendous amount of works was contributed by 4,799 authors. Among the top 10 scholars, Ian Colman took the first place in publication output (eight publications), followed by Serge Brande with seven papers. The difference in the number of publications by these authors was generally small but all top 10 authors possessed five works at the minimum. We made the author's cooperation network diagram by setting the co-authorship minimum number of documents of an author at 3, and 136 authors met the threshold. Moreover, we used overlay visualization to reflect the time span of these authors' products. Particularly, we normalized scores by subtracting mean and dividing by standard deviation (time span from −1 to 1). Judging from the distribution of cooperation situation presented in Figure 4A, the relationships among these authors were rather fragmented. It seemed that the scale of cooperation among scholars was relatively small, and there was still a lack of connection in the whole.

Meanwhile, 28,478 co-cited authors were identified in the 975 articles. Among them, Kessler RC occupied the #1 ranking in 167 citation counts, followed by Kovacs, M, Radloef, LS, and Beck, AT with 122, 98, and 92 citations, respectively. These authors' quotation amounts all exceeded 90 which implied that they were active and favorable authors in the field of exercise-depression-teenager research. Cooperation between co-cited authors were analyzed for the excavations of underlying partnerships. We set the minimum number of citations of an author at 20, and finally 136 authors were included. As shown in Figure 4B, a network visualization of co-cited authors was generated by nodes and links, and the larger node and thicker link represented closer cooperation. From the citation network, we saw that key authors were mostly in the position of crucial nodes and they pointed out the directions of research trends.

In detail, several scholars have played an important role in promoting research progress. In 2000, Sallis et al. (43) probed into the correlates of physical activity of young groups and examined the efficacy of physical activity and exercise as a universal prevention for depression in youth groups. Half a decade later, Penedo and Dahn (44) reviewed the relationship between exercise and well-being, and implied that exercise and physical activity can lead to better quality of life and health outcomes, including reducing symptoms of both depression and anxiety. In 2009, Babiss and Gangwisch (45) proved that sports participation can be used as a protective factor against depression and suicidal ideation by activating self-esteem and increasing social support. One year later in 2010, Brand et al. (46) conducted a comparative study between athletes and controls to investigate whether chronic vigorous exercising was related to improved psychological functioning (depressive symptoms, stress, state, and trait anxiety), and his results found that adolescents engaging in an enormous number of exercises positively related to favorable sleep patterns and psychological functioning. A 2013 survey from Kremer et al. (47) inquired into the relationship among physical activity, leisure-time screen use, and depression in youth groups, and reported that higher levels of exercise combined with lower levels of leisure-time screen use were relevant to lower depressive symptoms. In 2018, Bailey et al. (48) summarized the previous randomized controlled trials and did a meta-analysis-based review to establish the treatment efficiency of physical activity for depressive disorders in adolescents, and suggested that although physical activity seemed to be a promising and acceptable method for young populations with depression, further clinical effectiveness trials were still required to verify these findings.

### References Cluster Analysis

**Figure 5:**
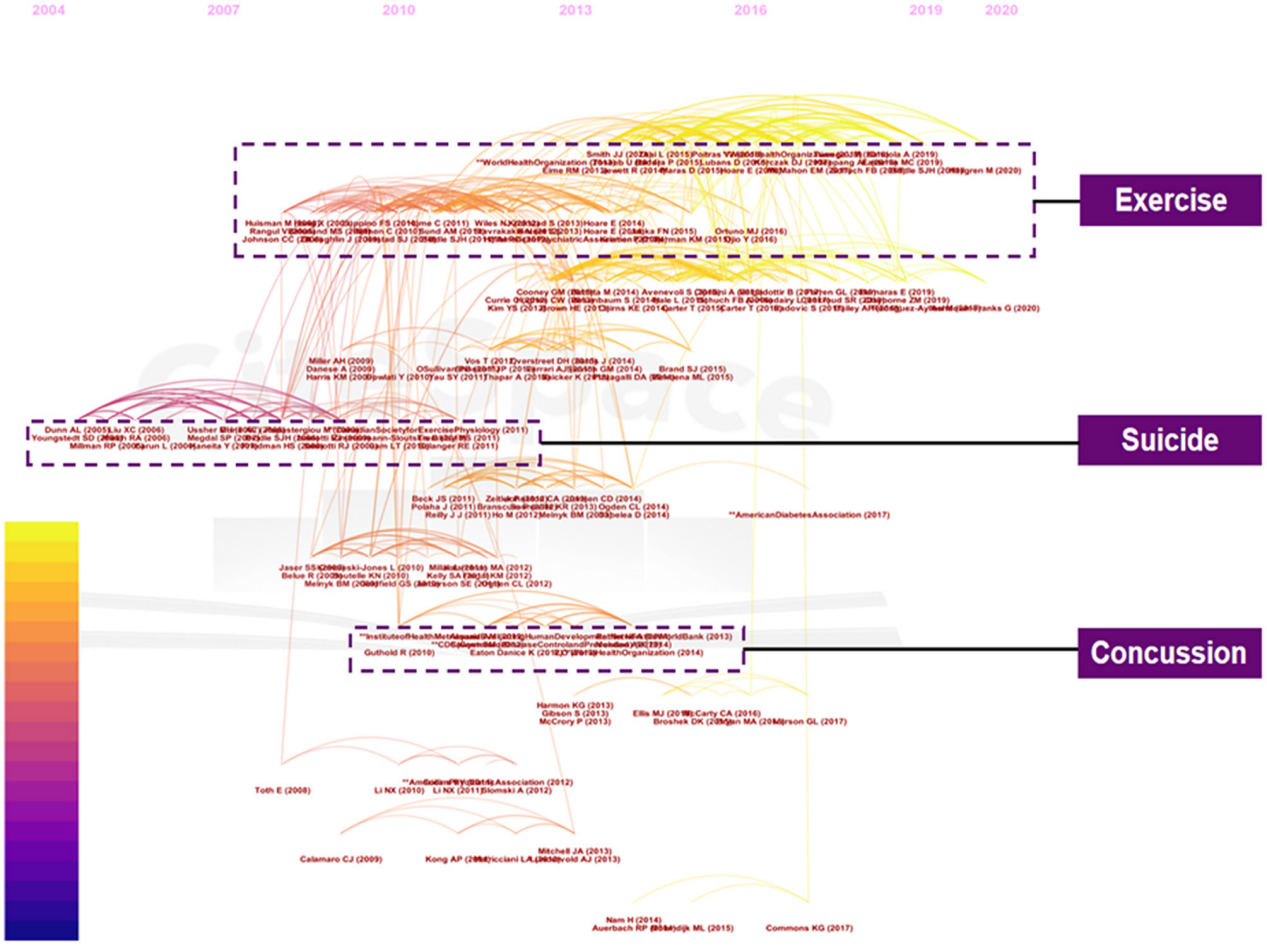
The timeline view of the knowledge map based on reference co-citation analysis of the exercise-depression-teenager field from 2000 to 2020.

References and relevant co-citation data were retrieved to generate major clusters, and knowledge domains of clusters were then mapped to seek research hotspots in the field of exercise-depression-teenager research. Table 3 lists the top 10 co-cited references, which were considered as great contributors in this area. In CiteSpace, there are three optional functions which can be applied to create label clusters: Log-Likelihood Ratio (LLR), Latent Semantic Indexing (LSI), and Mutual Information (MI). We used the Log-Likelihood Ratio strategy, because the creator of the CiteSpace software suggested that the LLR algorithm performed best when covering the “uniqueness and coverage” of all labels. On the basis of the reference cluster analysis, we found that current research focuses on the exercise-depression-teenager field. The research topics were divided into several clusters and then their time development trajectory was described, as shown in Figure 5. The earliest nodes were colored with purple and laid on the left side, whereas the most recent ones were printed with yellow and set at the right-hand position. Here, we introduced the following three noteworthy topics by keywords classification: “exercise,” “suicide,” and “concussion.” The modularity value (*Q* value) and weighted mean silhouette value (*S* value) were regarded as the evaluation strategies to assess the standard of clustering, and it was generally considered that a *Q* > 0.5 cluster and *S* > 0.7 meant that the cluster was convincing. In Figure 5, the *Q* equaled to 0.869 and *S* equaled to 0.921, which further verified the rationality of this clustering strategy.

The “exercise” cluster went from nearly 2010 to the present. Exercise intervention is mainly based on the theory of kinematics, neuron-development, and sports psychology. As an emerging method of depression countermeasures, exercise intervention did not get enough attention in the early years, but with more and more patients with depressive symptoms benefitting from sports and exercise, clinicians began to pay attention to the effect of exercise therapies. Depression can cause mental and psychological trauma to adolescent groups, thereby leading to homeostasis imbalance and central nervous system disorder, which is an extremely terrible portent for teenagers who are in a critical period of growth and development. At the molecular level, relevant studies (49–54) have also suggested that the concentration of noradrenaline and 5-hydroxytryptamine (5-HT) in the cerebrospinal fluid of sufferers with depression were significantly lower than that of healthy groups. In line with these effects, exercise was associated with various neurological improvements which might contribute to prevent or improve depression (55–58). Besides, recent literature (59) showed that the cellular changes induced by exercise were long-lasting, regardless of whether muscles became atrophic or not, which stood up for the viewpoint that sports-related activities can exert positive long-term results.

“Suicide” was another topic worthy of concern, which appeared mainly in the first decade of the twenty first century. Suicide risks originating from depression are of increasing concern on school campuses nowadays (60–62). Early studies already detected that depression was one of the leading risk factors for suicide (63), accompanied by substance abuse, adverse life events, separate family conditions, troubled relationships, and other problems (64, 65). There were also concerns (66, 67) about the dubious effects of traditional anti-depression drugs on the long-term development of teenagers, since major depression medication might further aggravate the social isolation of these teenagers, and even lead to suicide. When it comes to exercise activities decreasing suicide risks caused by depression, in 2003, Tomson et al. (68) assessed the relationship of playing sports outside of school, and of meeting health-related fitness indexes, to symptoms of depression (including correlations with thoughts of suicide and attempted suicide), suggesting that daily physical activities would both prevent depressive disorders and help young people with symptoms of depression to establish excellent health habits. A survey (69) conducted in 2007 also showed that when comparing high school active students with sports team non-participants, the odds of suicide ideation were lower among students frequently participating in vigorous-intensity physical activities. Several cross-sectional studies with teenagers (ages 11–25) further proved that exercise and sports might possess a protective nature against depressive disorders (45, 70–74), suicide attempts, and suicide (69, 72, 74–77). In brief, the interaction found between exercise and suicide risks suggested that increased sport activities could act as a protective, and preventive approach against suicide. Encouraging adolescents to be involved in more active sports can benefit them mentally and physically.

The “concussion” cluster was distributed mostly in the years between 2010 and 2015. Concussion is a form of traumatic brain injury and pediatric concussions are increasing with respect to prevalence rates. Youth groups tend to take even longer and require more effort to recover from depression caused by concussion than adults. A pilot study of concussed teenagers with symptoms over 1 month showed that post-concussion conditions including depression can be remarkably improved after an active recovery method including graded exercise (78). On the one hand, exercise has a positive effect on the improvement of depression and concussed symptoms. As an old Chinese proverb says: “The water that bears the boat is the same that swallows it up,” on the other hand, antagonistic or intense exercise-induced concussion is a special issue that affects thousands of children and adolescents annually in North America (79, 80), so excessive or incorrect exercise strategies are also potential risks for concussion, which may further lead to depression. Previous studies from pediatric and high school athlete cohorts have implied an upward trend of concussions in certain events like football or soccer (81–84). Evaluating clinical symptoms in the early stage and establishing corresponding clinical diagnostic procedures are two crucial means to reduce the potential risk of post-concussion syndrome including depression or anxiety (85–87). For the rehabilitation of this targeted group, preliminary evidence supported the combination of symptom-limited aerobic exercise, and corresponding physical therapy (88). In a nutshell, this cluster mainly focused on exercise intervention in the treatment of concussion-related complications and how to treat and prevent concussion risks caused by inappropriate exercise.

### Keywords Analysis

**Figure 6:**
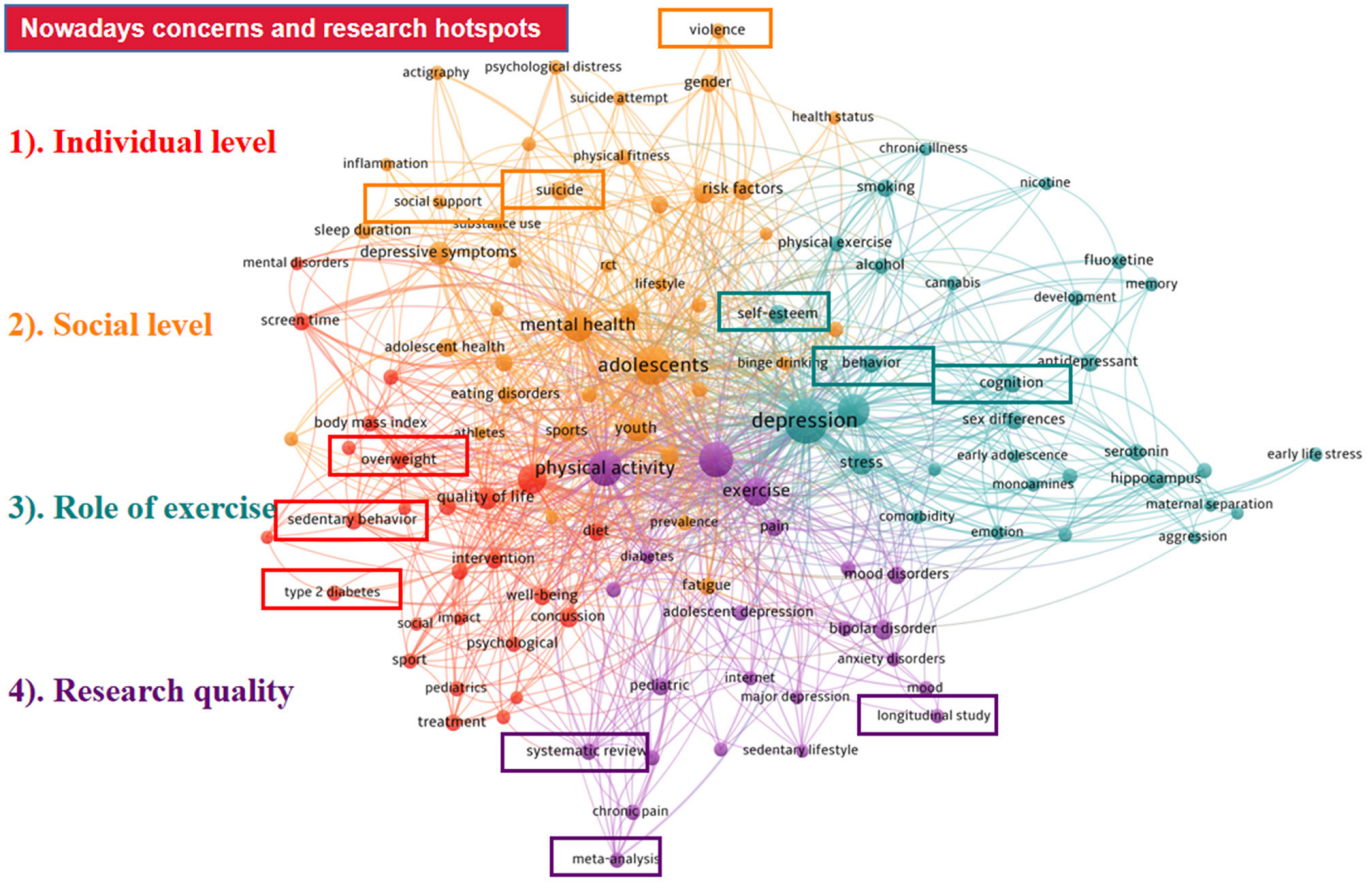
The distribution of the co-occurrence network of keywords in exercise-depression-teenager research.

**Figure 7:**
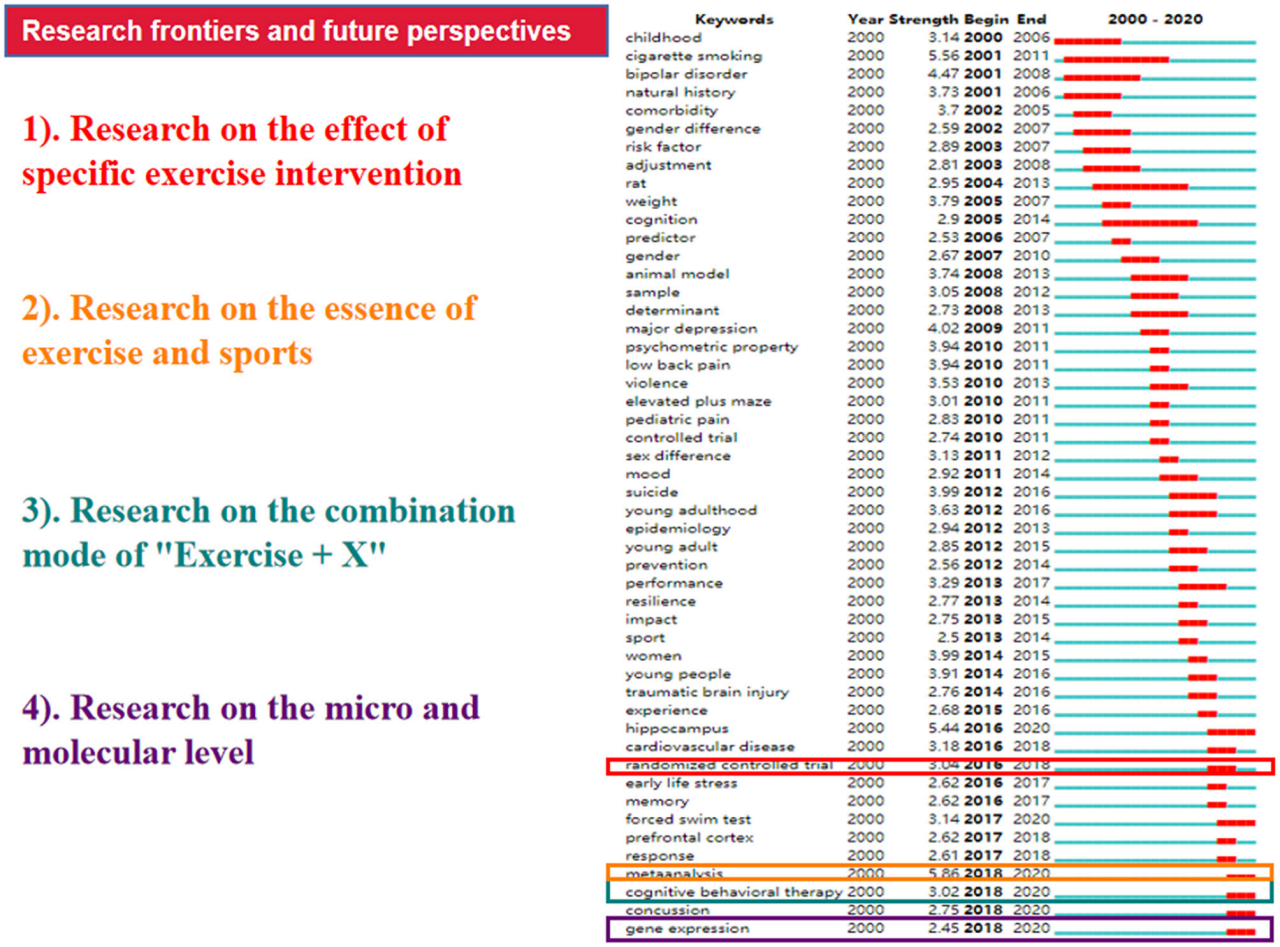
Top 50 keywords with the strongest citation burst in exercise-depression-teenager research.

Figure 6 (created by VOSviewer) presents the co-occurrence network of keywords within the scope of inclusion. The connections among all keywords are displayed with the thickness of the links representing the occurrence frequency and the node colors standing for the cluster classification. Among all keywords included, “depression” was the most frequently referred to keyword, with 343 co-occurrences, followed by adolescent (335 times), children (293 times), physical activity (194 times), and anxiety (157 times). In this figure, we set the minimum co-occurrence criteria at 4 as per the recommendation provided by VOSviewer. As a consequence, a total of 121 frequently referred keywords were incorporated and 922 links were built among them. Four clusters were then formed with different colors, and the bright red, sunset yellow, photo blue, and violet purple represent the following four clusters respectively: “individual level,” “social level,” “role of exercise,” and “research quality.” The top 50 keywords with strong burst strength in exercise-depression-teenager research are presented in Figure 7. The keywords with bursts lasting until 2020 included “hippocampus,” “forced swim test,” “meta-analysis,” “cognitive behavioral therapy,” “concussion,” and “gene expression,” which reflected the most recent research trends. The exploration of these keywords will be discussed in depth in the next section.

## Discussion

Here, we focused on the further detection of the co-occurrence and burst analysis of the major keywords. The generation of clustering was based on the information of the main keywords' co-occurrence. According to the classification of the causes of depression, we divided it into two categories: the individual factors of teenagers and the external factors caused by society. On the basis of the whole process of depression management, it is necessary for us to discuss the essential roles of exercise. At the same time, most of the current studies on exercise intervention in depression have expressed concerns about the research objects and quality, so it is meaningful to make a general summary based on this. Based on the above discussion of the four clusters, we have a profound grasp of the present concerns and research hotspots in this field.

1. Individual level: Keywords including “sedentary behavior,” “overweight,” “type 2 diabetes” can be considered as the teenagers' endogenous factors of depression. Teenagers who are overweight, physical inactive, have an irregular diet, smoke, and other characteristics are more likely to suffer from depression. However, these teenagers could prevent and alleviate the symptoms of depression by adjusting schedules, trying a reasonable diet, and performing regular exercise. Current findings have suggested that exercise or physical activities might be protective against depression caused by these series of factors (89–91), while the exercise type, disorder category, sport intensity, and intervention period all have influence on the effectiveness of exercise intervention. Thus, the different endogenous factors leading to depression still need to be further studied and should be personalized.
2. Social level: “Social support,” “suicide,” “violence,” and other relevant keywords can represent the main mechanisms of social factors on adolescent depression. Social factors can be divided in two ways: direct and indirect effect. Direct effect emphasizes that social support has a positive impact on individuals whether they are in a stress state or not. Indirect effect focuses on reducing the negative impact of stress events on individuals and decreasing the probability of individual depression caused by stress events. Taking adolescent depression caused by concussion as an example, the occurrence of such factors is usually not determined by the individual's will. While the attitudes of society toward young concussed patients are likely to affect the trend of depression development. It is significant to reduce social discrimination, physical violence, and language aggression for the recovery of such depressive symptoms. A sound social support structure, health education system, and regular organization of sports activities cannot only buffer the impact of negative emotions on teenagers and reduce the tendency of suicide, but also enable them to have better interpersonal relationships, so as to promote their mental health development.
3. Role of exercise: Terms containing “self-esteem,” “behavior,” and “cognition” were associated with the role of exercise. We divided the role of sports into two aspects: the physiological and psychological part. The physiological promotion of exercise on adolescents with depressive symptoms is to shape their body, and the main psychological improvement is to cultivate mental toughness and self-confidence. Exercise participation plays a positive role in keeping teenagers at a healthy body mass index (BMI) and releasing their stress. Except for improving physical ability like aerobic capacity of adolescents, exercise can also help shape mental toughness, a keyword which has also occurred frequently lately. Mental toughness is regarded as a cognitive strength variable comprising a series of values, attitudes, and emotions that enable an individual to negotiate with challenges and adversities (92), which is also especially explored within sport contexts. A study performed in 2012 (93, 94) focusing on adolescent cricketers discovered that the mental toughness profiles in this group were associated with negative emotional conditions, and cricketers with high levels of mental toughness reported lower levels of negative emotional states such as depression, anxiety, and stress. In line with this, mental toughness not only belongs to the realm of elite sport, but can also be applied in normal adolescents. One research (94) conducted in 2017 invited 1,361 teenagers to participate in exploring the associations among physical activity, subjective sleep, mental toughness, and other indexes, showing that greater physical activity was associated with more favorable subjective sleep, higher mental toughness, and lower depression. To sum up, exercise activities have a positive mind-body impact on depressive teenagers.
4. Research quality: Keywords including “systematic review,” “meta-analysis,” and “longitudinal study” were related to risk factors and research quality of studies focusing on exercise intervention in treating depression among teenagers. Several factors such as sample size, gender, age, family background, education level, countries, and regions may affect the accuracy of the results of exercise intervention on depression, especially gender difference, since a large number of studies have indicated that the effect of exercise treatment on depression was significantly different between boys and girls. Factors mentioned above are the main reasons for the different conclusions of studies with variations in designs, comparisons, and controls. Therefore, systematic reviews and meta-analyses have sprung up in recent periods to evaluate the effect size of exercise therapy on depression. Besides, there is still great demand for more high-quality, well-controlled reviews to provide suggestions for strategy exploration and clinical decision-making.

The bursts of the top keywords can be used to analyze evolution and future trends. In view of Figure 7, we can explore research perspectives and predict frontrunners. A total number of 50 prevalent keywords and cutting-edge fields of exercise-depression-teenager research were found and grouped into four categories:

1. Research on the effect of specific exercise intervention: Since numerous studies have clearly indicated a positive association between sports participation and psychological health, what kind of exercise is common and effective? At the beginning of the century, Larun et al. (95) suggested that sport might be an important instrument in improving the emotional health of children and adolescents, while there was little difference between high-intensity and low-intensity exercise. With the deepening and development of research, several articles and systematic reviews (48, 72, 96) reported that aerobic exercise might be considered as a promising strategy, and aerobic-based activity of moderate-to-vigorous intensity, engaged in multiple times per week over 8 or more weeks, could be recommended as best candidates for improving depression among teenagers (48). From a way to look good to an opportunity to feel good, aerobic exercise may help teenagers change their motivations for daily activity, which can improve their body satisfaction and self-esteem, and then reduce their depressive symptoms. Additionally, Chinese traditional exercise like Tai Chi might be an applicable strategy to alleviate the progression of depression in youth groups (97, 98). As a mindfulness-based exercise, Tai Chi can not only strengthen the body, but also help depressive teenagers build confidence and relieve anxiety. In addition, accumulating evidence has suggested that team sports are effective in diminishing risk and promoting rehabilitation for depressive symptoms (99–101). Besides, there is debate on whether high-intensity interval training (HIIT) is an appropriate type of exercise for adolescents with depression. One study showed that HIIT was able to enhance focus and reduce impulsive thoughts, which might improve adolescents' response to mental health treatment (102). However, some researchers argued that HIIT was too arduous and could evoke experiences of incompetence, failure, and lower self-esteem, thus reducing treatment effects (103). Furthermore, we considered that combat or collision sports like boxing, full-contact martial arts, rugby, ice hockey, or lacrosse should not be recommended as interventions for depressive symptoms. To summarize, moderate-intensity aerobic exercise and physical activities involving group participation could be recommended as feasible options for depressive adolescents, while the detailed intervention dose and strategy need to be verified by more high-quality RCTs.
2. Research on the essence of exercise and sports: By classifying different types of exercise and discussing their outcome indicators, current meta-analysis-based studies have discussed the nature of sports intervention. For the influence of exercise as a treatment for depressive teenagers, some previous literature concluded exercise as an alternative intervention. However, if we only regard exercise as a complementary strategy, it seems that one important point has been ignored. From the perspective of evolution, human beings were born to exercise, and our ancestors established cooperative relationships and social networks in exercise and physical-related activities such as planting, hunting, migrating, etc. However, in modern society, with a sedentary lifestyle due to the popularization of electronic products, over-nutrition caused by unhealthy diets, self-centered value on account of excessive family pampering, and exhaustion derived from academic pressure, children and teenagers gradually lost the drive to build a connection with society and became lonely, anxious, even depressive. The most essential value of sports activities to prevent or treat depression may be rooted in making these young people understand how to live with dignity, value, and meaning, as well as regain their connection with society. Hence, in this study, we summarized that the essence of exercise and sport is to help depressive adolescents “pick back [up] lost things.” However, the exploration of the sociology of physical exercise and essence of sports are still in pressing need to detect clear lines of demarcation between exercise intervention and depression treatment.
3. Research on the combination mode of “exercise + X”: Obviously, the strategy to treat depression is not just exercise. What effect will be achieved if more than one intervention mode is applied? Regular exercise combined with healthy diet and peaceful sleep are considered as important parts of complementary and alternative medicine in treating depression. Converging epidemiological evidence suggests that a sedentary lifestyle and lack of physical activity may lead to numerous chronic diseases. As shown in the burst keywords list, cardiovascular disease was also highly mentioned in recent years and it is not realistic to rely on only exercise intervention to solve these problems. Therefore, the treatment mode of combining various methods with exercise as the main line may be the best way to treat adolescent depression and its complications. Studies focusing on exercise intervention accompanied with lifestyle cures and cognitive behavior therapy should be further investigated to identify which mode is available for most targeted groups, as well as which intervention should be the first choice.
4. Research on the micro and molecular level: In recent years, a growing body of scholars have used neuro-physiology, genes, and omics analysis to explore the key pathways and factors to improve depression from the perspective of animal models. Current literature has proved that exercise influences the association of physiological and biochemical variables (104, 105), including serum levels of copper (Cu), zinc (Zn), serotonin, and salivary cortisol, which can achieve similar effects with antidepressants. Studies also initially showed that exercise and physical activity plays an analogous role as medicine *via* increasing brain neurogenesis in the hippocampus (106, 107). During the recovery stage of depression, exercise can induce a series of physical and emotional changes, including the release of endorphins and neurotransmitters, improving brain hemodynamics, enhancing nerve cell growth and synaptic plasticity, and reducing inflammation (108–110). In addition, the latest evidence revealed that physical exercise in the adolescence period could also serve as a predictor of depressive symptoms (111, 112). However, the metabolic crosstalk and the molecular mechanism of exercise used to treat depression are still not completely clear, and more regulatory factors and circulatory pathways are needed to prove adaptations induced by exercise intervention.

There were some limitations in the present study. First, the database analyzed in our research was limited to the Science Citation Index Expanded of WoSCC, and we did not include, data from other relevant search engines. However, the WoSCC SCI-E database has been recognized for the quality of its papers, and this database has been widely used for the retrieval of nature science publications. Second, this study generated a linguistic bias considering we only considered English publications, despite English remaining the most commonly applied language for publishing academic documents worldwide. Additionally, we only selected articles and reviews for analysis, the identified types of research may not fully represent all exercise-depression-teenager studies. Nonetheless, articles and reviews were the mainstream types of publications.

## Conclusions

This study conducted a bibliometric and comparative analysis from 2000 to 2020 and provided a bird's-eye view of exercise intervention in treating depression among teenager groups. We highlighted the opportunities and developments of exercise therapy applied in the treatment of adolescents' depression in an objective and systematic manner, which can not only assist scholars in extracting hidden information for further research, but also provide them with valuable directions for topic selection activities. The first part of this study examined the document trends, active authors, journals, countries, and institutions. The latter part was applied to perform cluster and burst analysis of the major keywords to identify research hotspots and explore potential topics in this field. As a cost-effective, space-saving, and more enjoyable component in the prevention and treatment of depression, exercise therapy was particularly promising in teenager groups, not only for its influence on physiological indexes, but also the shaping of personality and the return of social relations. More specifically, aerobic-based activities combined with team sports might be optimal exercise strategies for depressive youth. Finally, we considered the following four novel topics: “research on the effect of specific exercise intervention,” “research on the essence of exercise and sports,” “research on the combination mode of ‘exercise + X',” and “research on the micro and molecular level,” which should receive further attention in the coming years.

## Data Availability Statement

The original contributions presented in the study are included in the article/supplementary material, further inquiries can be directed to the corresponding author/s.

## Author Contributions

YY helped to organize the study, prepared datasets, performed the statistical analysis, and drafted the manuscript. DW conducted study design and prepared datasets. YW helped to prepare the datasets. ZL contributed to study design. XM contributed to study organization and revision of the manuscript. All authors read and approved the final manuscript.

## Conflict of Interest

The authors declare that the research was conducted in the absence of any commercial or financial relationships that could be construed as a potential conflict of interest.
